# Polymer–Colloid Complexes Based on Cationic Imidazolium Amphiphile, Polyacrylic Acid and DNA Decamer

**DOI:** 10.3390/molecules26082363

**Published:** 2021-04-19

**Authors:** Darya A. Kuznetsova, Dinar R. Gabdrakhmanov, Denis M. Kuznetsov, Svetlana S. Lukashenko, Valery M. Zakharov, Anastasiia S. Sapunova, Syumbelya K. Amerhanova, Anna P. Lyubina, Alexandra D. Voloshina, Diana V. Salakhieva, Lucia Ya. Zakharova

**Affiliations:** 1 FRC Kazan Scientific Center, Russian Academy of Sciences, Arbuzov Institute of Organic and Physical Chemistry, Arbuzov str. 8, 420088 Kazan, Russia; dashyna111@mail.ru (D.A.K.); Nemezc1988@yandex.ru (D.R.G.); kuznetsov_denis91@mail.ru (D.M.K.); notassl@yandex.ru (S.S.L.); anastasiya.strobykina@iopc.ru (A.S.S.); syumbelya07@mail.ru (S.K.A.); aplyubina@gmail.com (A.P.L.); sobaka-1968@mail.ru (A.D.V.); 2 Kazan National Research Technological University, Karl Marx str., 68, 420015 Kazan, Russia; zakharov_vm@mail.ru; 3 Institute of Fundamental Medicine and Biology, Kazan (Volga Region) Federal University, Kremlyovskaya St. 18, 420008 Kazan, Russia; DiVitSai@gmail.com

**Keywords:** imidazolium, amphiphile, polyacrylic acid, oligonucleotide, complexation

## Abstract

The solution behavior and physicochemical characteristics of polymer–colloid complexes based on cationic imidazolium amphiphile with a dodecyl tail (IA-12) and polyacrylic acid (PAA) or DNA decamer (oligonucleotide) were evaluated using tensiometry, conductometry, dynamic and electrophoretic light scattering and fluorescent spectroscopy and microscopy. It has been established that PAA addition to the surfactant system resulted in a ca. 200-fold decrease in the aggregation threshold of IA-12, with the hydrodynamic diameter of complexes ranging within 100–150 nm. Electrostatic forces are assumed to be the main driving force in the formation of IA-12/PAA complexes. Factors influencing the efficacy of the complexation of IA-12 with oligonucleotide were determined. The nonconventional mode of binding with the involvement of hydrophobic interactions and the intercalation mechanism is probably responsible for the IA-12/oligonucleotide complexation, and a minor contribution of electrostatic forces occurred. The latter was supported by zeta potential measurements and the gel electrophoresis technique, which demonstrated the low degree of charge neutralization of the complexes. Importantly, cellular uptake of the IA-12/oligonucleotide complex was confirmed by fluorescence microscopy and flow cytometry data on the example of M-HeLa cells. While single IA-12 samples exhibit roughly similar cytotoxicity, IA-12–oligonucleotide complexes show a selective effect toward M-HeLa cells (IC_50_ 1.1 µM) compared to Chang liver cells (IC_50_ 23.1 µM).

## 1. Introduction

Interactions between oppositely charged surfactants and synthetic polyelectrolytes are currently the focus of modern research [1,2,3,4,5,6]. This is mainly due to the possibility of the control of the properties of these systems by the variation of the chemical structure, ratio and concentration of components, molecular weight of the polymer, pH of the medium, temperature and ionic strength [7,8,9]. In addition, the fabrication of complexes can be mediated by different mechanisms, including the hydrophobic effect, van der Waals interactions and hydrogen bonding, with the contribution of dominating electrostatic interactions [10,11,12,13]. For these reasons, mixtures of polyelectrolytes and oppositely charged surfactants are characterized by the complicated behavior of solutions [14,15,16]. The addition of a high-molecular-weight component can change the aggregation thresholds of the system [17,18,19,20]. Moreover, the investigation of polymer–colloid systems allows simulating the interaction of cationic amphiphiles with natural polymers such as DNA, proteins and so on [7,21,22,23]. This is of importance in gene therapy [24,25] because the application of naked DNA molecules is complicated due to their huge size, the electrostatic repulsion between nucleotides and negatively charged cell membranes and the immune response observed [26]. Therefore, efficient transfection can be achieved using optimized carriers of polynucleotides, which allow compacting large DNA molecules and recharging them. This allows genetic material to be recognized by the cell [27,28], pass through the cell membrane by the endocytosis mechanism [29] and receive protection from biodegradation. From this point of view, cationic surfactants are among the most prospective carriers [30,31,32,33,34], including those bearing an imidazolium moiety [35,36,37,38]. Imidazolium amphiphiles and DNA were documented to form sustainable nontoxic complexes that protect from enzymatic degradation and provide a charge density sufficient for DNA transport through the cell membrane [35]. Nonviral vectors based on the mixture of lipids and imidazolium amphiphiles were reported and were less effective than commercially available lipofectamine 2000; however, they had lower toxicity [36].

**Figure 1:**
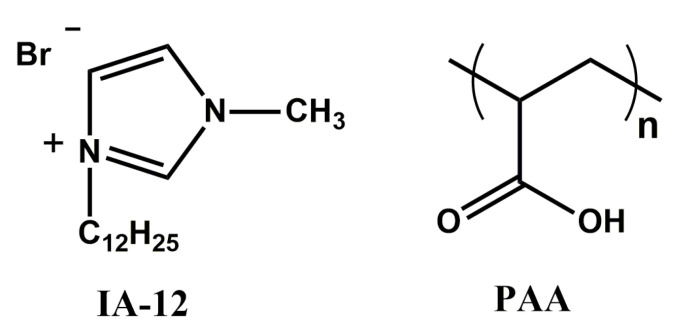
Chemical formulas of IA-12 and PAA studied in this report.

A brief literature overview makes it obvious that the fabrication of complexes based on amphiphiles and polyelectrolytes of various types is urgent. Nevertheless, despite the long-term study of these systems, there is little understanding of the factors influencing their functional activity. Much attention is paid to the interaction of cationic surfactants with anionic synthetic polyelectrolytes because they could simulate complexation between DNA and amphiphilic compounds. Unlike strong polyelectrolytes, weak polyelectrolytes demonstrate complicated and tunable solution behavior, which is controlled by the charge density of a polymer chain, which, in turn, is determined by the nature of macromolecules, concentration of charged additives, solution pH, etc. For cationic surfactant–polyacrylic acid (PAA) binary systems, the critical ionization degree α_c_ should be taken into account. The theoretical value of α_c_ = 0.35 can be calculated based on Manning’s counterion condensation theory [39]. Typically, electrostatic interactions prevail above α_c_, while the hydrophobic effect in combination with hydrogen binding contributes to complexation below α_c_. In accordance with the literature data [40], for surfactant–PAA systems, this threshold occurs at a pH of ca. 4.2, beyond which the ionization degree markedly increases and electrostatically driven complexes are formed. Based on this information, it can be expected that these complexes show stimuli-responsive properties regulated by the solution pH, concentration of components, ionic force, etc. This allows controlling the aggregation capacity and functional activity of the systems based on PAA. Therefore, this study is devoted to the elucidation of the self-assembling behavior and practical potentiality of the systems based on imidazolium amphiphiles bearing a dodecyl hydrophobic tail (IA-12, Figure 1) and two polyanions: (i) synthetic polyelectrolyte (polyacrylic acid, PAA, Figure 1) and (ii) a DNA decamer (oligonucleotide). For amphiphile/polyelectrolyte, systems their aggregation characteristics in aqueous solutions were quantified under different surfactant concentrations and fixed polyelectrolyte concentrations in accordance with an earlier protocol [41].

## 2. Results and Discussion

### 2.1. Mixed Systems Based on IA-12 and Polyacrylic Acid

Binary IA-12/PAA systems were studied by the variation of the amphiphile concentration and three fixed concentrations of polyelectrolyte (1, 3 and 5 mM). In accordance with our previous reports [41,42], a spontaneous pH of ~4 occurs under these conditions, which corresponds to an ~8% degree of polyelectrolyte ionization. This means that PAA can be specified as a weak polyelectrolyte bearing a negative charge.

**Figure 2:**
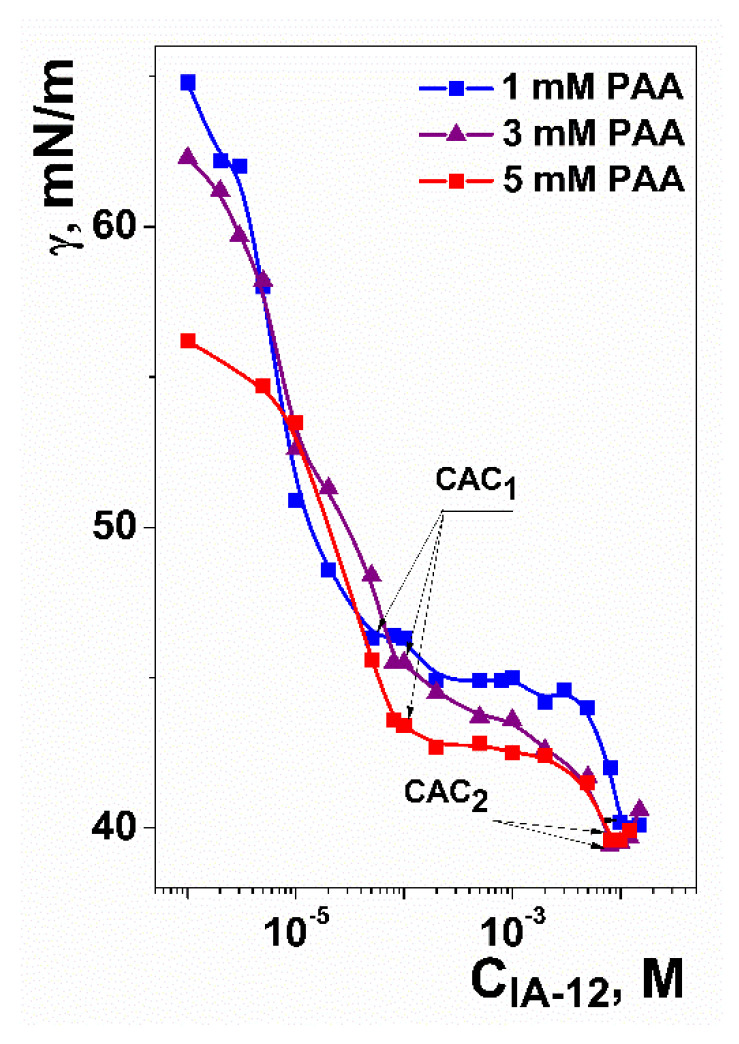
Surface tension isotherms for the IA-12/PAA binary systems at three fixed PAA concentrations; pH = 4, 25 °C.

The first stage of the investigation was dedicated to the study of the aggregation properties of polymer–colloid systems. For this purpose, the surface tension technique was successfully used (Figure 2). Inflection points in surface-tension isotherms are usually taken as the critical micelle concentration (CMC) or the critical association concentration (CAC). Usually, there are more inflection points in the case of surfactant/polyelectrolyte systems in comparison with individual amphiphile solutions. This depends on various factors, including the nature and concentration of components, molecular weight of the polymer, geometry of the molecules, solution pH, polyelectrolyte ionization degree, ionic strength, etc. It was shown that two inflection points were observed for the IA-12/PAA binary systems (Figure 2) regardless of the PAA concentration. The first inflection point (CAC_1_, see arrows in Figure 2) reflects the IA-12 concentration, at which the formation of amphiphile/polyelectrolyte complexes begins. The second inflection point (CAC_2,_ see arrows in Figure 2) corresponds to the saturation of macromolecules by amphiphile aggregates, after which individual IA-12 aggregates are formed. The CAC_1_ and CAC_2_ values obtained are compared with the CMC value for the individual IA-12 solutions [43] (Table 1). The addition of polyelectrolyte results in the formation of aggregates at a ca. 200 times lower amphiphile concentration (0.046 mM) than in the case of individual amphiphile solutions (9 mM). This effect is mostly expressed in the case of the lowest PAA concentration (1 mM) and diminishes with an increase in the PAA concentration (Table 1). This fact is probably due to the distribution of amphiphiles between different macromolecules with an increase in their amount in solution.

To obtain reliable information on the aggregation behavior of IA-12/PAA binary systems, they were examined by two additional techniques: conductometry and fluorescence spectroscopy using pyrene as a probe (Figures S1 and S2, Supplementary Materials). This is motivated by the fact that these methods are based on different physical principles responsible for their accuracy while providing specific advantages and limitations. Tensiometry evaluates the structural behavior of the systems at the surface layer rather than in the bulk phase. Meanwhile, the interfacial characteristics of polyelectrolyte–surfactant systems can be modulated by different factors that are not related to the aggregation. At the same time, conductometry provides a valuable instrument for the quantification of the aggregation behavior of charged species. The fluorimetry technique is very sensitive to changes in micropolarity that result from the surfactant aggregation. Data of the two alternative techniques are consistent with those obtained by tensiometry (Table 1).

**Figure 3:**
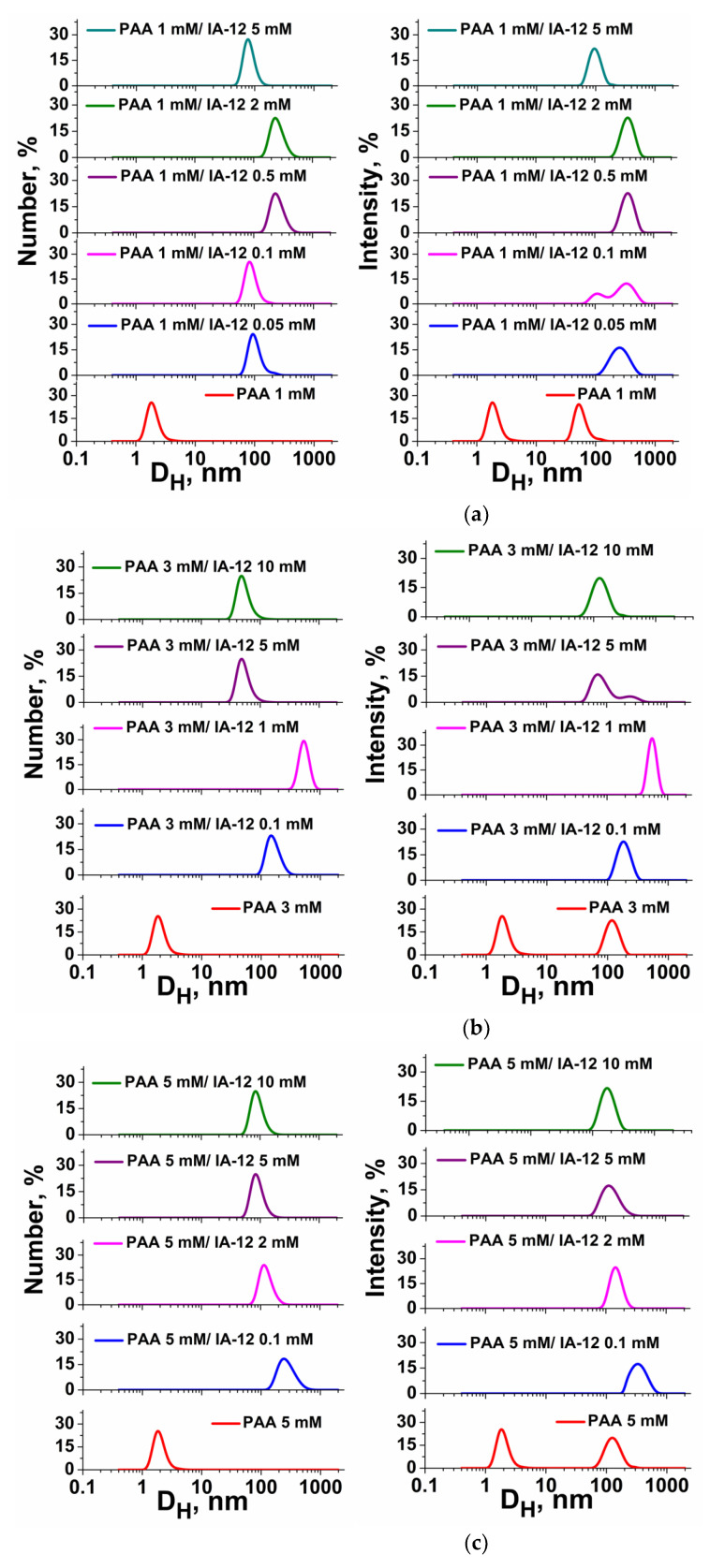
Number- and intensity-averaged size distribution for the IA-12/PAA binary systems at various fixed PAA concentrations: (**a**) 1 mM; (**b**) 3 mM; (**c**) 5 mM; 25 °C.

The size characteristics of the IA-12/PAA binary systems were evaluated using the dynamic light scattering technique for amphiphile concentrations beyond CAC_1_ (Figure 3). This method allows characterizing the hydrodynamic diameter D_H_ of mixed aggregates and proposes their morphology in aqueous solutions. As can be seen, large particles with a D_H_ of 100–150 nm are formed, which can increase to 350 nm with the amphiphile concentration in some cases. Earlier, we reported that the hydrodynamic diameter of individual amphiphile aggregates is 4–6 nm, and they most likely have a spherical shape [43]. Taking into account the small size of the particles in the individual PAA solutions (D_H_ = ~2 nm), it should be suggested that aggregates with a size of ≥100 nm are formed in mixed IA-12/PAA systems due to electrostatic attraction forces. Analogous trends in the variation of *D*_H_ with the amphiphile concentration are observed for all three fixed PAA concentrations. First, the aggregate sizes increase with the surfactant concentration, whereafter a decrease in the sizes occurs (Figure 3). The largest size of complexes probably reflects the agglomeration of IA-12 and PAA macromolecules close to the isoelectric point. The comparison of the number-averaged size distributions with intensity-averaged data reveals that good agreement occurred in the majority of systems (Figure 3). However, bimodal intensity-averaged size distributions could be seen in some cases, indicating an increase in the polydispersity of the system.

Additional information can be obtained through the analysis of correlation functions (Figures S3–S5). Aggregates of a smaller size show faster diffusion; hence, decay of the correlation function to the baseline occurs for a shorter period of time. In the case of the larger aggregates, the time of decay increased. For the IA-12/PAA 1 mM binary system (Figure S3), the maximum time of the decay of the correlation function (about 2000 µs) was found at 0.5 and 2 mM amphiphile concentrations. This corresponds to the largest aggregates in this system (Figure 3a). Moreover, the shape of the correlation function provides evidence of the existence of only one type of aggregate in the polymer–colloid solutions. This trend is observed for all the IA-12/PAA binary systems studied (Figures S3–S5).

**Figure 4:**
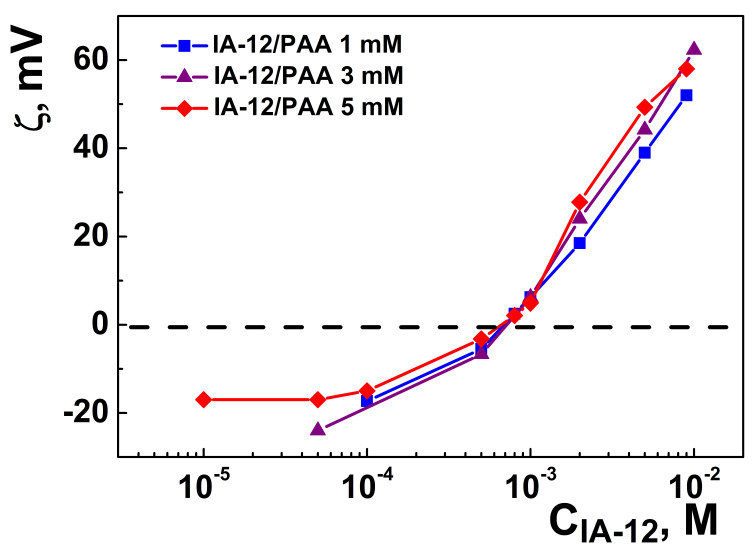
Zeta potential versus IA-12 concentration plot for the IA-12/PAA binary systems; 25 °C.

**Figure 5:**
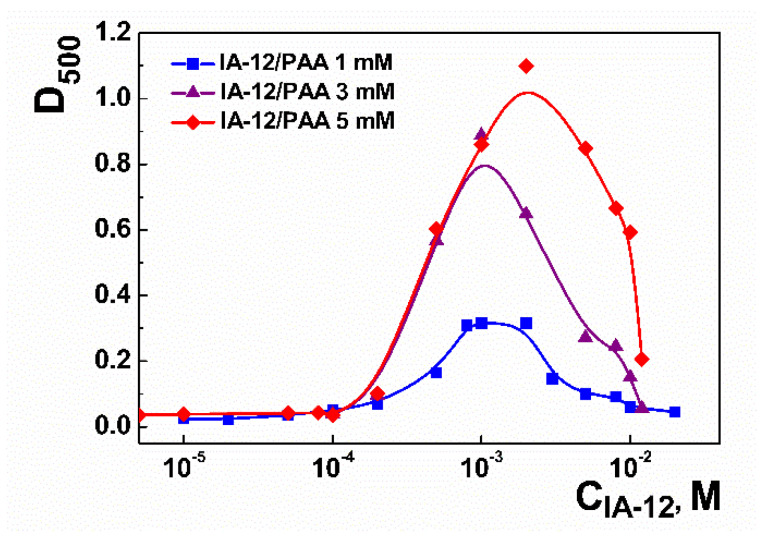
Optical density at λ = 500 nm versus IA-12 concentration plot for the IA-12/PAA binary systems; C_PAA_ = 1, 3, 5 mM, 25 °C.

The charge characteristics of the binary IA-12/PAA systems were evaluated using the electrophoretic light scattering technique. The data shown in Figure 4 and Figure S6 testify that an increase in the surfactant concentration is accompanied by a compensation of the negative charge of the PAA anion up to a zero value, after which positively charged systems appeared. Unlike the data available [42], the isoelectric point corresponding to the zero zeta potential of binary systems is practically unaffected by the PAA concentration. This can be due to several factors. First, PAA is a weak polyelectrolyte, and its charge behavior is strongly determined by the solution pH. Under spontaneous conditions, the solution pH is close to four (Figure S7). A slight decrease is observed with an increase in the surfactant concentration. The latter is probably caused by the shift of the pK_a_ value induced by micelles. Under these conditions, only~8% of carboxylic groups are ionized [41], which is probably responsible for the weak influence of the polyelectrolyte concentration on electrostatic interactions. Second, the charge behavior of the IA-12/PAA binary systems can be guided by a fine balance between several factors, including the variation in the solution pH as a function of the polymer and surfactant concentration and a shift in pK_a_ values. Therefore, no simple correlation is observed in this case. Third, additional information can be obtained from the turbidity data, demonstrating that binary systems tend to become inhomogenous close to an equimolar ratio (Figure 5); therefore, some discrepancy of data in this concentration range can occur.

In all cases, an increase in the IA-12 concentration results in the enhancement of the opalescence in the system followed by its disappearance, with a further increase in the micelle concentration (Figure 5). It is noteworthy that the turbidimetric data are in good accordance with the above variation in zeta potential in the systems. The highest absorbance was detected in the area of the isoelectric points of the systems, while the solutions were more transparent in the region of the existence of positive or negative zeta potential. This phenomenon could be caused by the fact that uncharged colloid species tend to aggregate, thereby inducing inhomogeneity. On the contrary, charged particles are more stable due to electrostatic repulsion preventing their aggregation and sedimentation. As can be seen, the maximum of absorbance shifts to the right with an increase in the PAA concentration, thereby indicating that the charge behavior of the binary systems is influenced by the polyelectrolyte concentration, although this is unobvious from the zeta potential data in Figure 4.

### 2.2. Amphiphile/DNA Decamer Interactions

**Figure 6:**
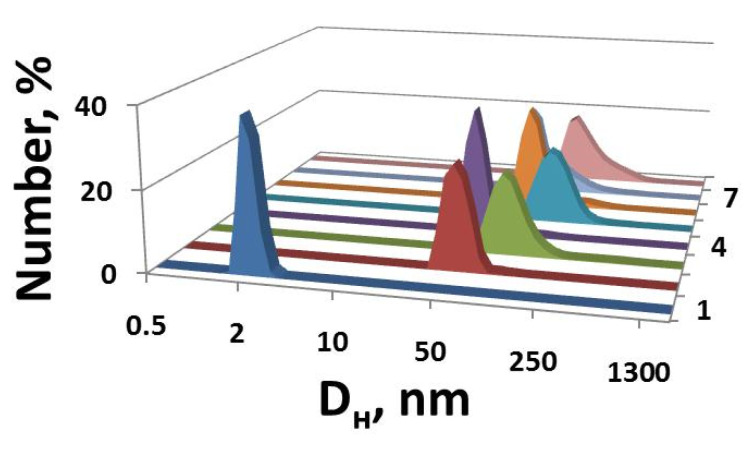
Number-averaged size distribution for the IA-12/oNu binary system at various amphiphile/oNu molar ratios: 1—individual oNu, 2—0.02, 3—0.045, 4—0.094, 5—0.194, 6—0.394, 7—0.79, 8—1.2; 9—3.9; 25 °C.

The next step of the study was dedicated to the interactions between the imidazolium-containing amphiphile and DNA decamer (oligonucleotide (oNu)). These investigations allow simulating the binding of IA-12 with nucleotide units of DNA. Initially, the size and charge characteristics of IA-12/DNA decamer complexes were evaluated, and the binding parameters of the components were quantified. To this end, methods of dynamic and electrophoretic light scattering and fluorescence spectroscopy were used. Dynamic light scattering was applied for the determination of the hydrodynamic diameter of particles in the IA-12/oNu binary system (Figure 6). As can be seen, binding of the components responsible for the complex formation is observed. This is evident from the enlargement of the D_H_ value of particles from 2 nm for an individual oNu to 50 nm and higher sizes (up to 250 nm) for the IA-12/oNu binary systems. It is noteworthy that these sizes are optimal for biotechnological purposes because they allow the nanoparticles to circulate in the bloodstream for a long time [44].

**Figure 7:**
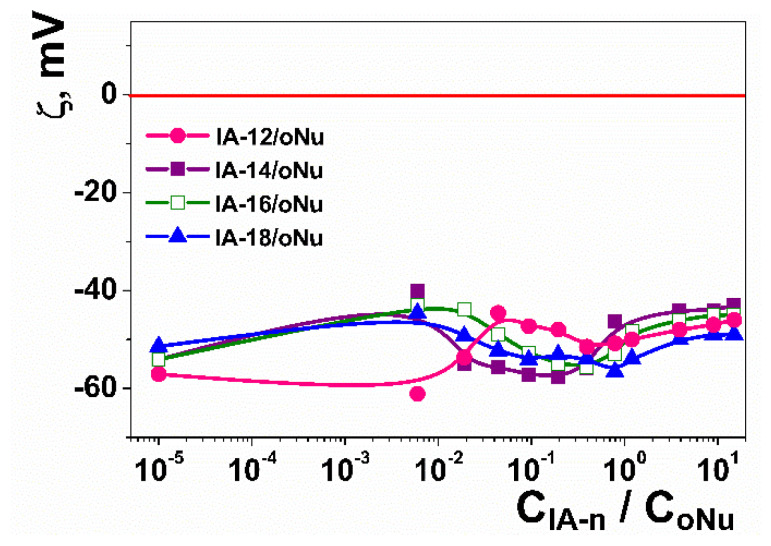
Electrokinetic potential versus amphiphile/oNu molar ratio for the IA-12/oNu, IA-14/oNu [45], IA-16/oNu [45] and IA-18/oNu [45] binary systems; 25 °C.

Another important criterion for the efficacy of potential nonviral vectors is their ability to neutralize the negative charges of nucleotide units, which was examined by the electrophoretic light scattering method (Figure 7). It was demonstrated that IA-12 has a weak ability of oNu charge compensation (Δξ does not exceed 20 mV); the isoelectric point was not achieved even with the excess of amphiphile. This trend has earlier been reported for the higher homologs of IA-12 [45], which was attributed to the fact that the delocalized positive charge of the imidazolium group cannot compensate for the negative charge of the phosphate backbones.

**Figure 8:**
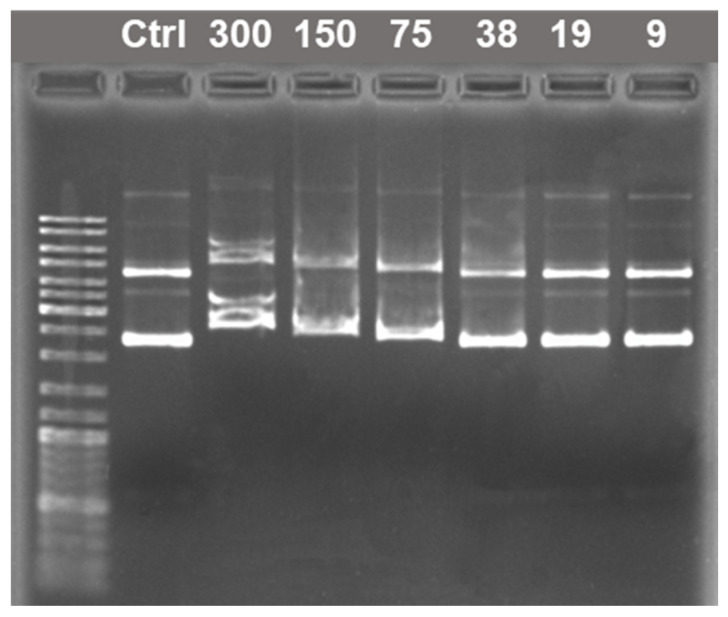
Electrophoretic mobility of pDNA in a complex with IA-12. Ctrl shows pure pDNA. N/P ratios are indicated above the corresponding wells. A DNA ladder from 100 to 10,000 bp was used.

To test whether a DNA oligomer can be used for the reliable prediction of the binding behavior of macromolecular analogs, electrophoretic analysis involving pDNA was carried out (Figure 8), which demonstrated that IA-12 does not bind with pDNA in a wide concentration range of the amphiphile. Some retardation of both supercoiled and relaxed forms of pDNA was, however, observed at increased N/P ratios from 38:1 to 300:1, indicating a weak interaction of the cationic surfactant with pDNA. The typical cationic surfactant CTAB used as a reference compound was shown to completely bind pDNA at an N/P ratio of 14:1 (Figure S9). The DNA-binding ability of CTAB was inferior to that observed for cationic polymers such as cationic polyaspartamides with an N/P ratio as low as 1:1 [46]. These data are in good agreement with the above zeta potential results of the IA-12/oNu complexes, testifying that electrostatic interactions play a minor role in the complexation. Unlike ammonium surfactants, imidazolium head groups with delocalized cationic charge give an insufficient contribution to phosphate anion neutralization.

DNA–surfactant complexes were characterized by the DLS technique at an N/P ratio of 10:1 (Figures S10–S12). CTAB formed with pDNA well-defined nanosized complexes (D_H_ 267 nm, PDI 0.234) with a highly positive zeta potential, indicating the condensation and cationization of pDNA by the surfactant. Under the same conditions, IA-12 neither packed pDNA nor induced its cationization, further showing the lack of its strong electrostatic interaction with pDNA and, therefore, the different DNA-binding properties of IA-12 compared to CTAB.

**Figure 9:**
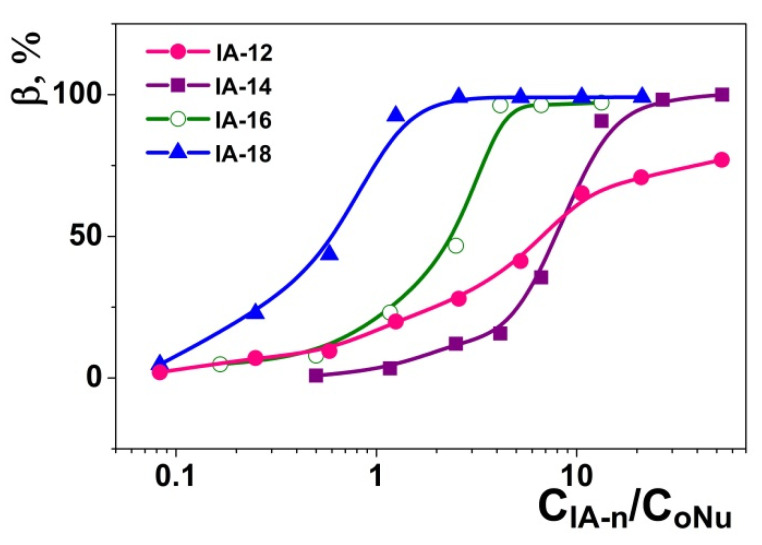
Binding degree of IA-12 with oNu versus IA-n concentration plot for the IA-12/oNu, IA-14/oNu [45], IA-16/oNu [45] and IA-18/oNu [45] mixtures; 25 °C.

The additional quantification of IA-12/oNu binding was carried out using the fluorescence technique and ethidium bromide (EB) as an intercalation probe. This approach is based on the exclusion of EB from the complexes by surfactant molecules, which is accompanied by the quenching of EB fluorescence. Emission spectra of EB/oNu complexes at various IA-12 concentrations (Figure S13) demonstrate that the higher the amphiphile concentration, the lower the intensity of the corresponding band. Fluorescence spectra for various IA-12/oNu molar ratios were used for the calculation of the IA-12/oNu binding degree *β* (Figure 9) using Equation (1). An increase in the amphiphile concentration is shown to induce an increase in the *β* value up to 75% under IA-12 excess, while no charge neutralization was observed in this range (Figure 7). It is noteworthy that earlier reported data for higher homologs of IA-12 (tetradecyl, hexadecyl and octadecyl derivatives) exhibited an almost quantitative binding of oNu [45]. This emphasizes the crucial role of the hydrophobic effect and cooperative interactions in the binding efficacy of imidazolium surfactants toward nucleotides. It can be proposed that an alternative intercalation mechanism of binding probably occurs apart from a weak electrostatic contribution. This is based on the fact that amphiphiles with a planar aromatic imidazolium head group are capable of locating nucleobases. Importantly, the alkyl tail length of imidazolium amphiphiles strongly determines their intercalation ability. In particular, homologs with shorter alkyl tails cannot interact with polynucleotides in such a way [47]. Comparative analysis of data obtained for imidazolium homological series allows us to conclude that the superposition of two factors controls the IA-12 complexation with a DNA decamer, namely hydrophobic binding and the intercalation mechanism.

**Figure 10:**
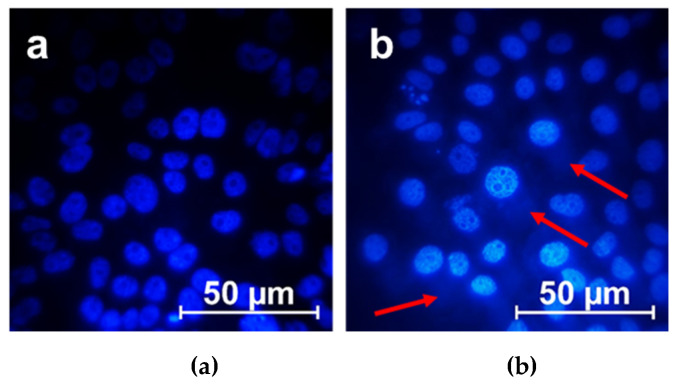
Fluorescent micrographs of HeLa cells in the absence (**a**) and in the presence of the IA-12/oNu complex (**b**); C_oNu_ = 1 mM, C_IA-12_ = 0.5 mM. The arrow indicates the presence of oligonucleotide in the cytoplasm of cells.

To test whether the IA-12/oNu complex could be transfected through the cell barrier to its interior, a fluorescence microscopy technique was used. Figure 10 demonstrates HeLa cancer cells stained with Hoechst 33342 in the absence and in the presence of the IA-12/oNu complexes. This dye is capable of binding with both DNA of the nucleus of HeLa cells (Figure 10a), and the added oNu marked them with blue. In Figure 10b, the blue nimbus corresponding to oNu localization is clearly seen close to the stained nucleus of the HeLa cell, which indicates that successful penetration of the complexes through the cell barrier occurred.

**Figure 11:**
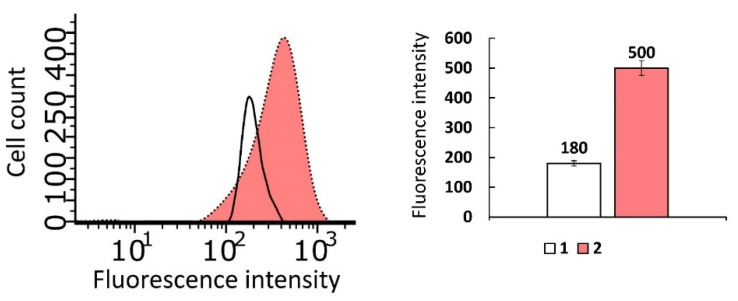
Cellular uptake study of the IA-12/oNu complex. 1—Intact M-HeLa cells; 2—M-HeLa cells in the presence of the IA-12/oNu complex.

The cellular uptake of the IA-12/oNu complexes was further testified using flow cytometry (Figure 11). Untreated cells were used as negative controls. After treatment with substances, M-HeLa cells were fixed and stained with Hoechst 33342 (blue), which was used as a fluorescent probe. The dye allows one to mark cell nuclei and oNu penetrated into cells in the form of blue spots. It was shown that upon treatment with IA-12/oNu complexes, the fluorescence intensity was higher than in the control, which indicates that the penetration of the test complexes into M-HeLa cells occurred.

From the viewpoint of a practical application, cytotoxicity is an essential characteristic of the systems. Table 2 summarizes the cytotoxic effect of the single IA-12 solution and IA-12/oNu complexes toward M-HeLa and Chang liver cell lines. Cationic surfactants are typically characterized by a rather high cytotoxicity, which is confirmed by the IC_50_ values of IA-12 given in Table 2, with a higher cytotoxic effect occurring against Chang liver cells. However, the transition to the binary IA-12/oNu complex is accompanied by the appearance of selectivity in cytotoxic action, namely a sharp decrease in IC_50_ values (to 1.1 µM), which occurs with cancer cells along with an increase in the viability of normal cells. Analogous changes in cytotoxic activity are documented in [48], where an MTT test revealed a decrease in the cytotoxicity of complexes compared to single cationic surfactants.

### 2.3. Membranotropic Properties and Cell Penetration

**Figure 12:**
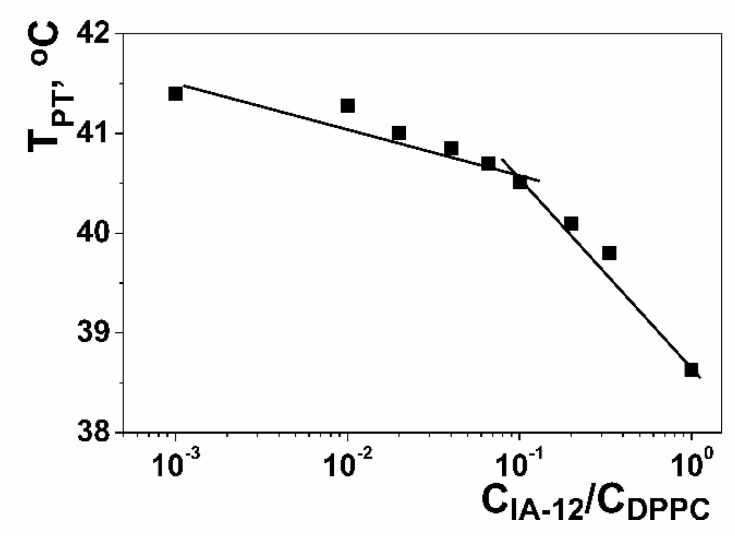
DPPC main phase transition temperature versus the amphiphile/lipid molar ratio plot for the IA-12/DPPC binary system.

Another important feature of drug and gene delivery systems based on amphiphilic compounds is their ability to pass through cell membranes composed of lipid bilayers. With this in mind, the ability of IA-12 to integrate with dipalmitoylphosphatidylcholine (DPPC) liposomes was examined. This property was studied using the turbidimetric titration of DPPC liposome dispersion by IA-12 solution to determine the temperature of the gel/liquid crystal phase transition. In the absence of foreign compounds, the temperature of the DPPC main phase transition (T_PT_) equals 41 ± 0.1 °C (Figure S14). Therein, T_PT_ is taken as the inflection point in reversed S-shape plots. Surfactants and other additives are capable of changing the T_PT_ value due to ordering or disturbing the lipid bilayer (Figure S14). Figure 12 visualizes turbidimetric data in terms of the temperature of the main phase transition versus the IA-12/DPPC molar ratio. The initial decrease in the T_PT_ value with the IA-12 concentration indicates the integration of amphiphile molecules in the lipid bilayer that resulted in the disordering of DPPC alkyl chains. This promotes an increase in the permeability of the lipid bilayer that could be used for biotechnological tasks. The slope of dependence became higher at C_IA-12_/C_DPPC_ ≥ 0.1, which can be attributed to the solubilization of liposomes in IA-12 aggregates.

## 3. Materials and Methods

### 3.1. Chemicals

Imidazolium amphiphile IA-12 was synthesized in accordance with the standard experimental procedure [45,49]. Commercially available double-stranded oligonucleotide (oNu) with 10 nucleotide units in each strand (GCGTTAACGC, molecular weight is 3028 g/mol, Joint Stock Company Syntol, Moscow, Russia) was used. Plasmid DNA (pDNA) pVax1-DsRed (3665 bp) was kindly gifted by Dilara Gatina (Omics Technology Laboratory, Kazan Federal University, Kazan, Russia). Agarose for molecular biology, ethidium bromide and hexadecyltrimethylammonium bromide (≥99.0%) were purchased from Sigma-Aldrich. As probes for fluorometric measurements, ethidium bromide (Sigma-Aldrich, St. Louis, MO, USA, 95%) and pyrene (Sigma-Aldrich, St. Louis, MO, USA, 99%) were used. Lipoid PC 16:0/16:0 (1,2-dipalmitoyl-sn-glycero-3-phosphocholine, DPPC) was a gift from Lipoid GmbH (Ludwigshafen, Germany). Hoechst 33342 (Sigma-Aldrich, St. Louis, MO, USA, 99%) was applied for the fluorescence microscopy assay. Polyacrylic acid with an average molecular weight of 1800 g/mol (Sigma-Aldrich, St. Louis, MO, USA, 99%) was used. Polymer concentrations were given as a molar concentration on a monomer basis. Purified water (18.2 MΩ cm resistivity at 25 °C) from Direct-Q 5 UV equipment (Millipore S.A.S. 67120 Molsheim-France) was used for all solution preparation.

### 3.2. Preparation of Samples

Liposome preparation was carried out by the thin lipid hydration technique as follows [49,50,51]: a specific amount of DPPC (5.4 mg) was solved in 100 µL of chloroform. The solvent was removed by evaporation overnight at room temperature. The obtained lipid film was suspended by 1 mL of water and thermostated at 55–60 °C in a water bath for 30 min. The fabricated rude suspension was frozen and thawed 5 times using liquid nitrogen and a water bath (60 °C), respectively. After that, the dispersion was extruded 21 times by using a syringe extruder (LiposoFast Basic, Avestin, Ottawa, ON, Canada) through a polycarbonate filter with 100 nm pores.

A stock solution of a DNA decamer was prepared by the dissolvation of commercial crystalline oligonucleotide in 4 mM Tris–HCl buffer (pH = 8.0) and its dilution down to 10 mM (concentration per nucleotide unit). The mixture was heated at 95 °C for 5 min and immediately chilled in an ice bath.

### 3.3. Methods

#### 3.3.1. Tensiometry

A Krűss K06 tensiometer (Hamburg, Germany, Du Nouy ring detachment method) was used for measurements of the surface tension of the amphiphile/PAA binary systems. The volume of amphiphiles solutions was 10 mL. The sample was thermostated for 5 min at 25 °C before measurement. Detachment of the ring for each sample was repeated at least 3 times, and measurements with a deviation of ≤0.1 mN/m were taken into account.

#### 3.3.2. Conductometry

Specific conductivity was measured using an Inolab Cond 720 conductometer at 25 °C. The sample was thermostated for 5 min at 25 °C before measurement. Specific conductivity values with a deviation of no more than ±1 μSm/cm were taken into account.

#### 3.3.3. Fluorescence Spectroscopy

A survey of the emission fluorescence spectra of pyrene was performed using a Cary Eclipse G9800A fluorescence spectrophotometer (Agilent Technologies, Santa Clara, CA, USA) at 25 °C. The excitation wavelength was 335 nm. Emission spectra were registered in the range of 350–500 nm with a constant scanning speed (120 nm/min). A cuvette with a 1 cm width was used in all measurements. Fluorescence intensities of the first (*I*_I_) and third (*I*_III_) vibrational peaks of pyrene at 373 nm and 384 nm, respectively, were extracted from the collected spectra.

Fluorescence spectra of the oligonucleotide–ethidium bromide complexes were recorded in the range from 500 to 700 nm at an excitation wavelength of 480 nm. A 0.8 mL sample containing 0.5 µM of EB and a 10 µM concentration of oligonucleotide (counting on 1 nucleotide unit) in 4 mM Tris–HCl buffer (pH = 8.0) was equilibrated for 10 min at 25 °C. After that, a specific aliquot of the amphiphile solution was added to the mixture, and the fluorescence emission spectrum was registered [52,53]. Each concentration dependence was performed 2 times, and the standard deviation of the results was less than 2%. The following equation was used for the quantitative evaluation of the binding degree of oNu to amphiphile:

β = (*I*_bound_ − *I*_obs_)/(*I*_bound_ − *I*_free_) (1)

where *I*_free_ and *I*_bound_ are the intensities of fluorescence of free EB and probe bound to oNu, respectively; and *I*_obs_ is the fluorescence intensity observed during titration measurements at a specific amount of amphiphile added.

#### 3.3.4. Dynamic and Electrophoretic Light Scattering

Measurements of size and charge characteristics of samples were carried out using dynamic and electrophoretic light scattering techniques at Malvern ZetaSizer Nano ZS apparatus (Malvern Instruments Ltd., Malvern, UK). A source of irradiation was a He-Ne gas laser with 4 MW power (λ_operating_ = 633 nm). Collected signals were treated in terms of frequency and phase analysis of scattered light using software attached to the device. All measurements were carried out at a 173° scattering angle. Computation of particle size was performed in accordance with the Stokes–Einstein equation [54]:

D = kT/(6πηR)(2)

where k is the Boltzmann’s constant, T is the absolute temperature, η is the solvent’s viscosity and R is the hydrodynamic radius.

The zeta potential calculation was based on three measurements of electrophoretic mobilities using the Smoluchowski relationship:

ζ = μη/ε(3)

where ζ is the zeta potential, η is the dynamic viscosity of the solution, μ is the mobility of the particle and ε is the dielectric constant [55].

The hydrodynamic diameter and zeta potential of pDNA complexes with IA-12 and CTAB were measured on a ZetaSizer Nano ZS analyzer (Malvern Instruments) in a U-cuvette in 50 mM HEPES buffer (pH = 7). The final concentration of DNA and the N/P ratio were 10 μg/mL and 10:1, respectively, for both complexes.

#### 3.3.5. Electrophoretic Analysis

To obtain complexes, pDNA at a final concentration of 10 μg/mL was gently mixed with IA-12 (90–3000 μg/mL) or CTAB (5–160 μg/mL) in phosphate-buffered saline (PBS). The mixture was incubated at ambient temperature for 20 min. Electrophoretic analysis of pDNA–surfactant complexes was performed in 1% agarose gel in Tris-acetate–EDTA buffer using electrophoresis apparatus (Bio-Rad Laboratories, Hercules, CA, USA). Electrophoresis conditions were detailed previously [46]. Briefly, pDNA (200 ng per well) was separated at a voltage of 8 V/cm for 60 min and then stained with ethidium bromide (0.5 μg/mL). The gels were visualized using a ChemiDoc™ XRS Plus gel documentation system (Bio-Rad Laboratories, Hercules, CA, USA). O’Gene Ruler DNA Ladder Mix (100–10,000 bp) (Fermentas, Waltham, MA, USA) was used.

#### 3.3.6. Turbidimetry

The phase behavior of amphiphile/PAA binary mixtures was studied using a Specord 250 PLUS spectrophotometer (Analytik Jena, Jena, Germany). An aliquot of polyelectrolyte solution was added to IA-12 solution of a specific concentration. The mixture was kept for 2 min, and the optical density of the solution was measured at a 500 nm wavelength.

Phase transitions in amphiphile/DPPC mixtures were studied using a Specord 250 PLUS spectrophotometer (Analytik Jena, Jena, Germany). An optical density at a 350 nm wavelength as a function of the temperature was registered for the establishment of the lipid main phase transition in the IA-12/DPPC binary system. The temperature varied in the range from 35 to 45 °C. Primarily, a turbidimetric plot for individual DPPC liposomes (0.7 mM) was obtained. Further, an aliquot of 5 mM amphiphile solution was added to the liposome dispersion, and an analogous turbidimetric plot was registered. These operations were repeated for several added aliquots of amphiphile solution. Turbidimetric plots were fitted in accordance with the van ’t Hoff two-state model, which assumes that the inflection point in the turbidimetric plot corresponds to the main phase transition of DPPC.

#### 3.3.7. Fluorescence Microscopy

Hoechst 33342 dye (0.1 mg/mL) was used as a contrast agent to localize the oligonucleotide. This dye binds to double-stranded DNA with a preference for domains enriched in adenine and thymine. Experiments were carried out using a Nikon Eclipse Ci-S fluorescence microscope (Nikon, Tokyo, Japan) at a magnification of 1000× and by means of the multifunctional Cytell Cell Imaging system (GE Health Care Life Science, Umeå, Sweden) using Automated Imaging BioApp at a magnification of 1000×.

#### 3.3.8. Flow Cytometry Assay

M-HeLa cells at 1 × 10^6^ cells/well were seeded in six-well plates. After 24 h of incubation, the IA-12/oNu complex was added to the test wells at a concentration of 0.5 μM. The uptake of the IA-12/oNu complex cells was analyzed using flow cytometry (Guava easyCyte 8HT, Luminex Corporation, Austin, TX, USA). Untreated cells were used as negative controls.

#### 3.3.9. In Vitro Cytotoxicity Assay

The evaluation of the toxicity of the IA-12 and IA-12/oNu systems was estimated by means of the multifunctional Cytell Cell Imaging system (GE Health Care Life Science, Sweden) using the Cell Viability Bio App. Cell lines M-HeLa (epithelial carcinoma of the cervix, HeLa subline, M-HeLa clone) were acquired from the Type Culture Collection of the Institute of Cytology (Russian Academy of Sciences, Saint Petersburg, Russia) and Chang liver normal cells from the N.F. Gamaleya Research Center of Epidemiology and Microbiology (Moscow, Russia). The cells were seeded in a concentration of 10^5^ cells/mL in 96-well plates (Eppendorf, Hamburg, Germany), then 150 μL of standard Eagle’s medium (PanEco, Moscow, Russia) was added per well, and incubation proceeded with CO_2_ at 37 °C. The medium was then supplemented with 10% fetal calf serum and 1% nonessential amino acids. Twenty-four hours after seeding, the systems under study were added at preset dilutions, 150 μL to each well. The dilutions were prepared immediately in a nutrient medium. The experiments were repeated three times. Intact cells cultured in parallel with experimental cells were used as a control.

## 4. Conclusions

In this study, complexation in mixed systems based on imidazolium amphiphile IA-12 and poly- or oligomeric anions of synthetic and natural origin was demonstrated. The addition of polyacrylic acid to a single surfactant system markedly reduces the aggregation thresholds of the system by ca. 200 times and leads to the electrostatically driven formation of stable polymer–colloid complexes with a hydrodynamic diameter of 100–150 nm. Unlike synthetic polyelectrolytes, the complexation of IA-12 with oligonucleotide is not electrostatically mediated, as evidenced by weak charge neutralization demonstrated by the zeta potential study and gel electrophoresis experiments. At the same time, high IA-12/oNu complexation efficacy is testified by ethidium bromide quenching data. Turbidity measurements demonstrated the membrane tropic ability of the imidazolium surfactant, after which the cellular uptake of the IA-12/oNu complex was proved by fluorescence microscopy and flow cytometry data. Selective cytotoxicity of the complexes was observed toward M-HeLa cells with IC_50_ = 1.1 µM, while a markedly higher value of IC_50_ = 23.1 µM was determined for Chang liver cells.

## Figures and Tables

**Table 1:** Aggregation thresholds of the IA-12/PAA binary systems obtained by different physicochemical techniques.

C_PAA_, mM	Aggregation Thresholds (mM)			
	Tensiometry		Conductometry	Fluorimetry
	CAC_1_	CAC_2_		
0	9 [43]	-	10 [43]	9 [43]
1	0.05	10.0	5	0.05
3	0.08	9.0	4	0.05
5	0.10	8.0	3.5	0.07

**Table 2:** The cytotoxic effect of IA-12 in the individual system and in the binary system with oNu toward normal and tumor human cell lines.

IC_50_ µM			
M-HeLa		Chang Liver	
IA-12	IA-12/oNu	IA-12	IA-12/oNu
39 ± 0.1	1.1 ± 0.08	17.0 ± 0.1	21.3 ± 1.8

## Data Availability

Data are contained within the article.

## Supplementary Materials

The following are available online. Figure S1: Specific conductivity versus amphiphile concentration plot for individual IA-12 solutions and IA-12/PAA binary systems at fixed PAA concentrations; 25 °C. Figure S2: Intensity ratio of the first and third vibronic peaks of pyrene vs. amphiphile concentration for the IA-12/PAA binary systems at fixed PAA concentrations; 25 °C. Figure S3: DLS correlation functions for the IA-12/PAA binary system at various amphiphile concentrations and a constant PAA concentration; C_PAA_ = 1 mM; 25 °C. Figure S4. DLS correlation functions for the IA-12/PAA binary system at various amphiphile concentrations and a constant PAA concentration; C_PAA_ = 3 mM; 25 °C. Figure S5: DLS correlation functions for the IA-12/PAA binary system at various amphiphile concentrations and a constant PAA concentration; C_PAA_ = 5 mM; 25 °C. Figure S6: Zeta potential versus IA-12 concentration plot for the IA-12/PAA binary systems; 25 °C (the linear X-axis). Figure S7: pH changes in IA-12/PAA aqueous solutions; 25 °C. Figure S8: Molecular weight marker O’Gene Ruler DNA Ladder Mix. Figure S9: Electrophoretic mobility of pDNA in complex with CTAB. Ctrl shows pure pDNA, N/P ratios are indicated above the corresponding wells. A DNA ladder from 100 to 10,000 bp was used. Figure S10: Intensity-averaged size distribution for the IA-12/pDNA system; N/P ratio of 10:1; 25 °C. Figure S11: Intensity-averaged size distribution for CTAB/pDNA system; N/P ratio of 10:1; 25 °C. Figure S12: Zeta potential for IA-12/pDNA (green) and CTAB/pDNA (red) complexes; N/P ratio of 10:1; 25 °C. Figure S13. EB/oNu emission fluorescence spectra in the presence of various amounts of IA-12 (the arrow indicates the direction of the increase in the amphiphile concentration); 25 °C. Figure S14: Typical turbidimetric plot for DPPC liposomes in the absence and in the presence of IA-12 (the amphiphile/lipid molar ratio is 1:5), C_DPPC_ = 0.7 mM [56].

## Sample Availability

Samples of the compounds are not available from the authors.
