# Supplementary material for: Polymer–Colloid Complexes Based on Cationic Imidazolium Amphiphile, Polyacrylic Acid and DNA Decamer
Source: Molecules. 2021 Apr 19;26(8):2363. doi: 10.3390/molecules26082363 (PMC8072887; doi:10.3390/molecules26082363)
Supplement: Supplementary file 1 [file molecules-26-02363-s001.zip › molecules-1115804-supplementary.pdf]

Supplementary Information

Polymer-Colloid Complexes Based on Cationic Imidazolium Amphiphile, Polyacrylic Acid and DNA Decamer

Darya A. Kuznetsova [1], Dinar R. Gabdrakhmanov [1], Denis M. Kuznetsov [1], Svetlana S. Lukashenko [1], Valery M. Zakharov [2], Anastasiia S. Sapunova [1], Syumbelya K. Amerhanova [1], Anna P. Lyubina [1], Alexandra D. Voloshina [1], Diana V. Salakhieva [3] and Lucia Ya. Zakharova [1,*]

[1] Arbuzov Institute of Organic and Physical Chemistry, FRC Kazan Scientific Center, Russian Academy of Sciences, Arbuzov str. 8, 420088 Kazan, Russian Federation; dashyna111@mail.ru (D.A.K.); Nemezc1988@yandex.ru (D.R.G.); kuznetsov_denis91@mail.ru (D.M.K.); notassl@yandex.ru (S.S.L.); anastasiya.strobykina@iopc.ru (A.S.S.); syumbelya07@mail.ru (S.K.A.); aplyubina@gmail.com (A.P.L.); sobaka-1968@mail.ru (A.D.V.)

[2] Kazan National Research Technological University, Karl Marx str., 68, 420015 Kazan, Russian Federation; zakharov_vm@mail.ru

[3] Institute of Fundamental Medicine and Biology, Kazan (Volga Region) Federal University, Kremlyovskaya St. 18, 420008 Kazan, Russian Federation; DiVitSai@gmail.com

* Correspondence: luciaz@mail.ru (L.Y.Z.); Tel.: +7-(843) 273-22-93

1. Conductometry assay.

Investigation of IA-12/PAA binary system was performed using conductometry technique (Figure S1). It was shown, that the inflection point is observed in narrow concentration range for all three systems (3.5 – 5 mM). Since these values are between listed in Table 1 CAC_1 and CAC_2 values, it could be assumed, that they reflect the fabrication of single premicellar aggregates of IA-12 formed after saturation of PAA macromolecule.

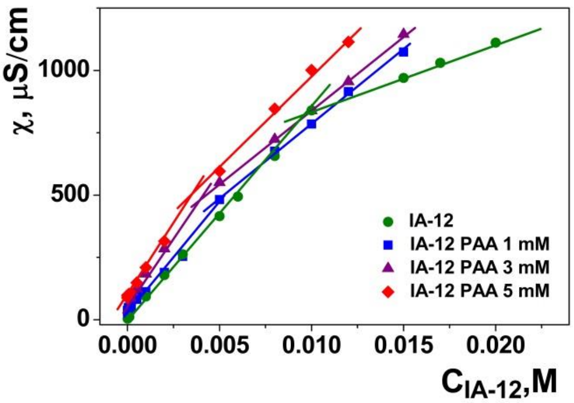

Figure S1. Specific conductivity versus amphiphile concentration plot for IA-12 individual solutions and IA-12/PAA binary systems at fixed PAA concentrations, 25 ^{o}C.

2. Fluorescence spectroscopy measurements (pyrene ratiometry).

Fluorescence spectroscopy using pyrene is another technique for detection of aggregation of amphiphiles in bulk solutions. It is widespread and well-known, since this hydrophobic probe has both high sensitivity and low operating concentrations (~ 1 μM). Pyrene is known to have five characteristic bands in emission spectrum. Intensity of the first and the third ones and their ratio (I_1/I_3) may characterize the micropolarity of the medium, that could be used for confirmation and characterization of microheterogeneous systems occurrence. In the case of all IA-12/PAA systems decrease of I_1/I_3 ratio due to the transition of hydrophobic probe from polar bulk phase into lipophilic interior of aggregates was demonstrated (Figure S2). Aggregation thresholds of systems could be

determined as points, after which I_1/I_3 values went to plateau [56]. Obtained values are listed in Table 1 and very close to CAC_2 values found by tensiometry technique.

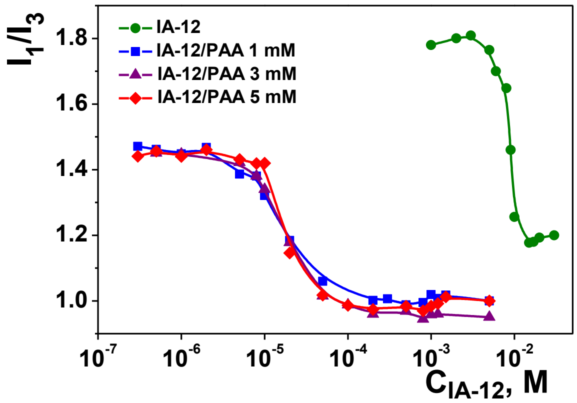

Figure S2. The intensity ratio of the first and the third vibronic peaks of pyrene vs amphiphile concentration for IA-12/PAA binary systems at fixed PAA concentrations; 25 °C.

S1. Aguiar, J.; Carpena, P.; Molina-Bolívar, J.A.; Carnero Ruiz, C. On the determination of the critical micelle concentration by the pyrene 1:3 ratio method. *J. Colloid Interface Sci.* **2003**, *258*, 116–122. https://doi.org/10.1016/S0021-9797(02)00082-6.

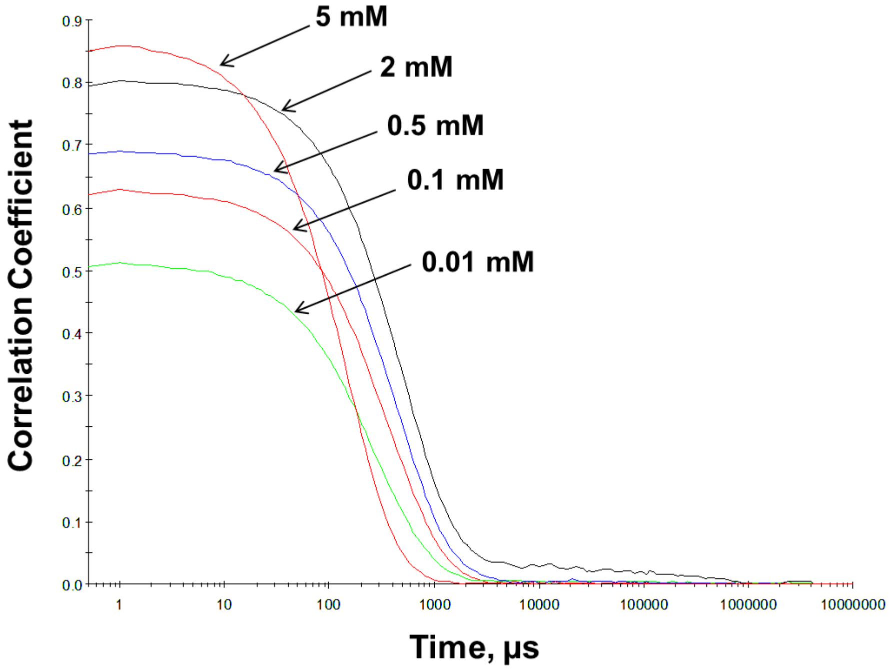

Figure S3. DLS correlation functions for IA-12/PAA binary system at various amphiphile concentrations and constant PAA concentration; C_{PAA} = 1 mM; 25 °C.

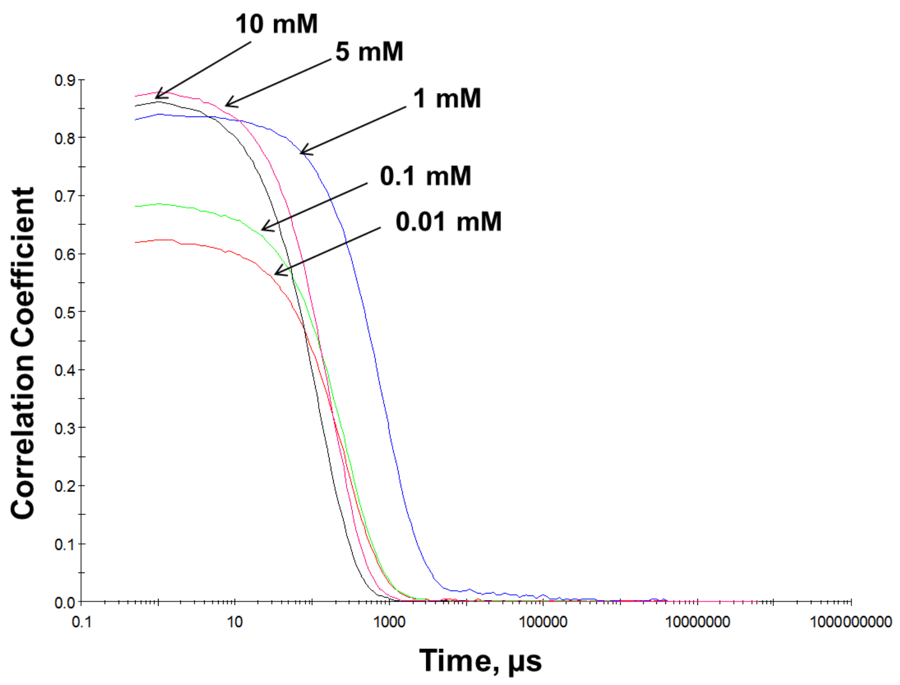

Figure S4. DLS correlation functions for IA-12/PAA binary system at various amphiphile concentrations and constant PAA concentration; C_{PAA} = 3 mM; 25 ^{o}C.

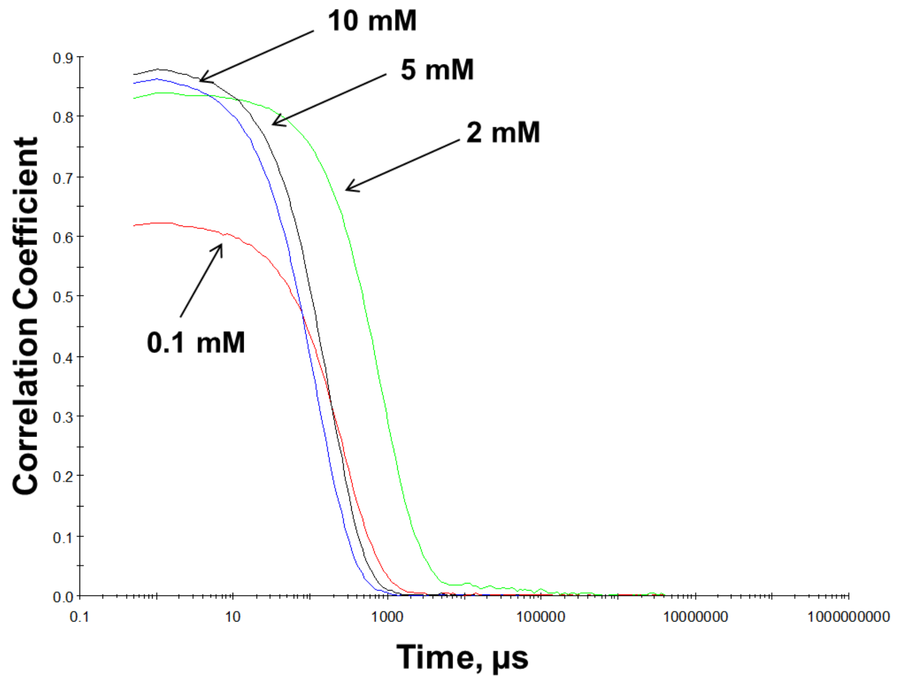

Figure S5. DLS correlation functions for IA-12/PAA binary system at various amphiphile concentrations and constant PAA concentration; C_{PAA} = 5 mM; 25 ^{o}C.

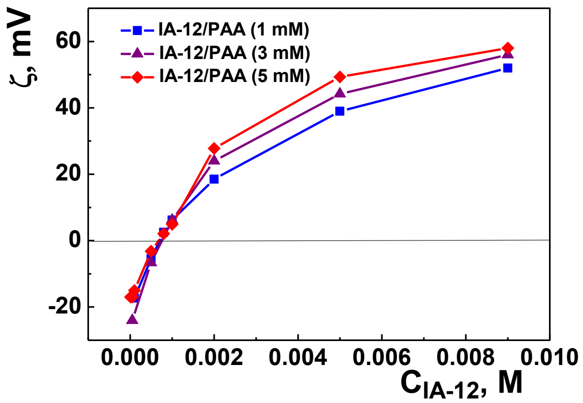

Figure S6. Zeta-potential versus IA-12 concentration plot for IA-12/PAA binary systems, 25 °C (the linear X-axis).

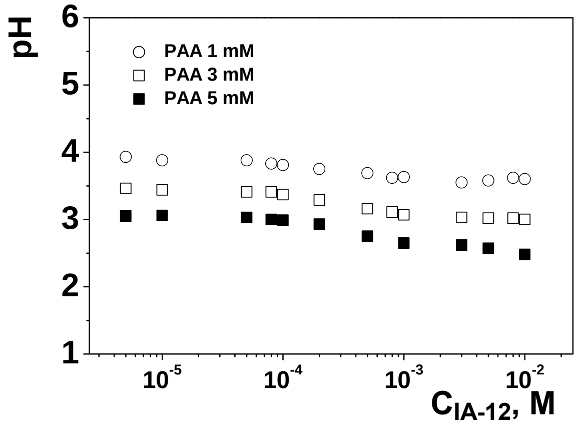

Figure S7. pH changes in IA-12/PAA aqueous solutions; 25 °C.

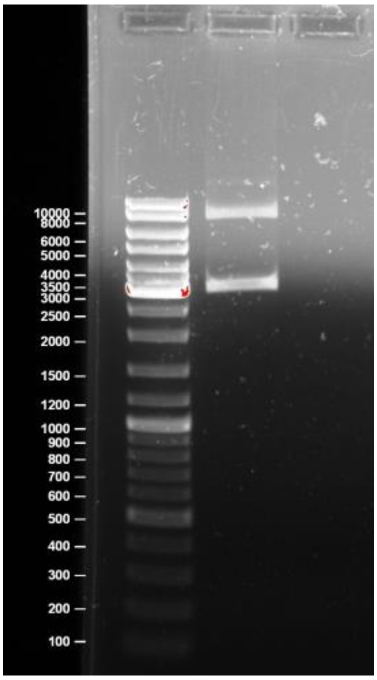

Figure S8. Molecular weight marker O'Gene Ruler DNA Ladder Mix.

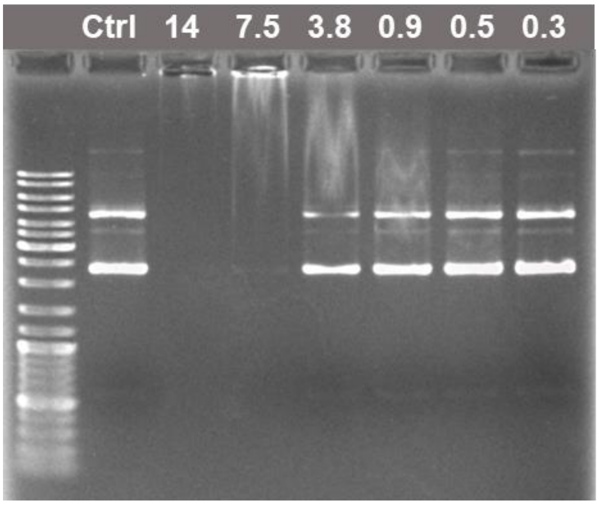

Figure S9. Electrophoretic mobility of pDNA in complex with CTAB. Ctrl shows pure pDNA, N/P ratios are indicated above corresponding wells. DNA ladder from 100 to 10000 bp was used.

			Size (d.nm):	% Intensity:	St Dev (d.nm):
Z-Average (d.nm):	665,6	Peak 1:	697,2	54,9	227,5
PdI:	0,600	Peak 2:	117,7	22,7	32,97
Intercept:	0,435	Peak 3:	5275	12,2	425,4
Result quality :	Refer to quality report				

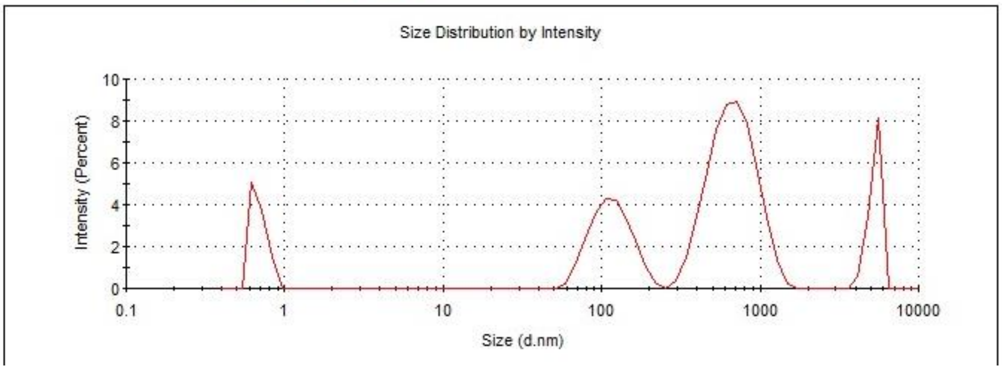

Figure S10. Intensity averaged size distribution for IA-12/pDNA system; N/P ratio of 10:1; 25 °C.

			Size (d.nm):	% Intensity:	St Dev (d.nm):
Z-Average (d.nm):	267,2	Peak 1:	355,7	100,0	190,5
PdI:	0,234	Peak 2:	0,000	0,0	0,000
Intercept:	0,940	Peak 3:	0,000	0,0	0,000
Result quality :	Good				

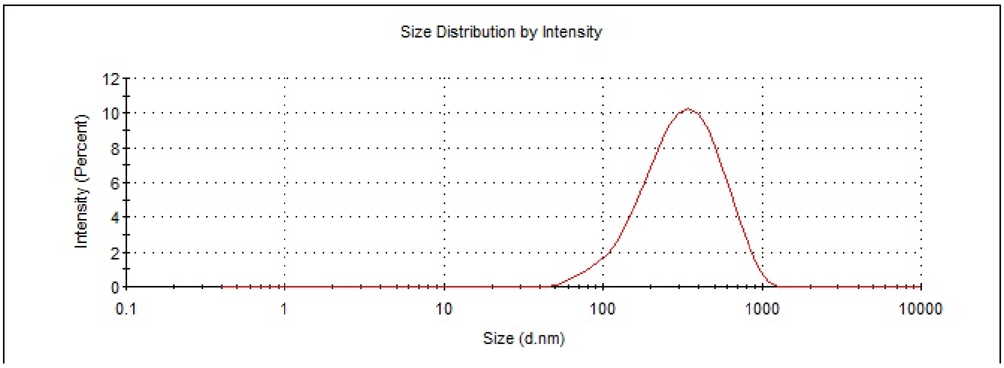

Figure S11. Intensity averaged size distribution for CTAB/pDNA system; N/P ratio of 10:1; 25 °C.

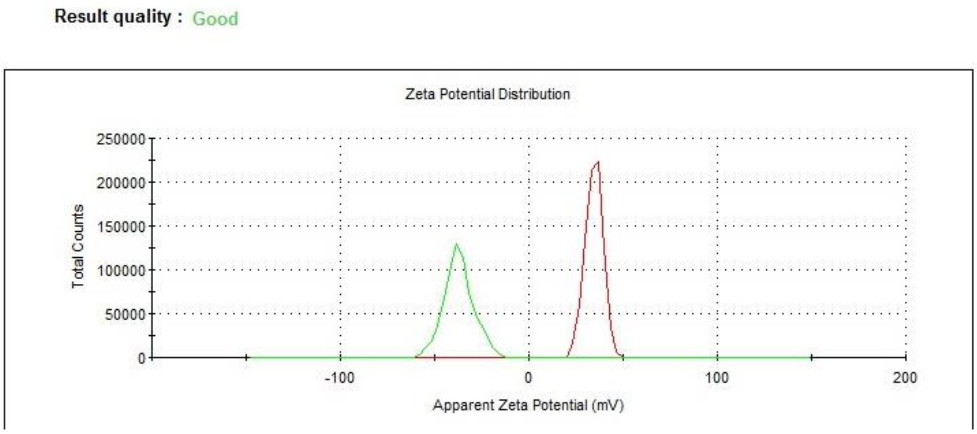

Figure S12. Zeta-potential for IA-12/pDNA (green) and CTAB/pDNA (red) complexes; N/P ratio of 10:1; 25 °C.

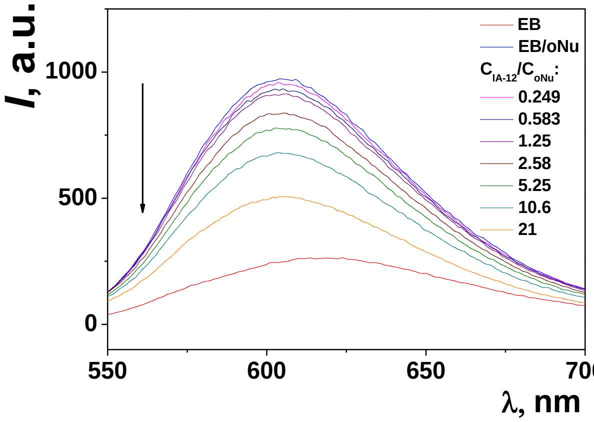

Figure S13. EB/oNu emission fluorescence spectra in the presence of various amounts of IA-12 (the arrow indicates on the direction of the increase of amphiphile concentration); 25 °C.

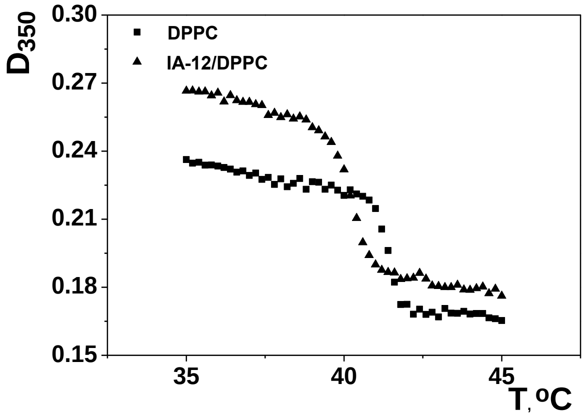

Figure S14. Typical turbidimetric plot for DPPC liposomes in the absence and in the presence of IA-12 (amphiphile/lipid molar ratio is 1:5), C_{DPPC} = 0.7 mM.
